# Ground Tire Rubber Modified by Ethylene-Vinyl Acetate Copolymer: Processing, Physico-Mechanical Properties, Volatile Organic Compounds Emission and Recycling Possibility

**DOI:** 10.3390/ma13204669

**Published:** 2020-10-20

**Authors:** Łukasz Zedler, Paulina Burger, Shifeng Wang, Krzysztof Formela

**Affiliations:** 1 Department of Polymer Technology, Faculty of Chemistry, Gdańsk University of Technology, 80–233 Gdańsk, Poland; lukasz.zedler@pg.edu.pl (Ł.Z.); paulina_anna_burger@interia.pl (P.B.); 2 Department of Polymer Science and Engineering, Shanghai Jiao Tong University, Shanghai 200240, China; shfwang@sjtu.edu.cn

**Keywords:** ground tire rubber, modification, ethylene-vinyl acetate copolymers, waste management, performance properties, recycling

## Abstract

Ground tire rubber (GTR) was reclaimed and modified with 10 phr of ethylene-vinyl acetate copolymer via low-temperature extrusion process. Processing, physico-mechanical properties, volatile organic compounds emission, and recycling possibility were investigated. In order to better understand the impact of used modifiers, their efficiency was compared with trans-polyoctenamer, which is an additive that is commercially dedicated to waste rubber recycling. The results showed that a relatively small amount of ethylene-vinyl acetate copolymer improves the mechanical properties of modified reclaimed GTR and also allows further recycling by multiple processing without the deterioration of performance after three cycles.

## 1. Introduction

Three-dimensional networks present in rubber goods improve their mechanical properties, chemical resistance, and thermal stability. On the other hand, these excellent performances cause difficulties in waste rubbers management and further recycling [1,2,3].

The mainstream of waste rubbers is end-of-life tires. Gerrard and Kandlikar [4] proposed a breakdown of materials used in a passenger vehicles, which indicated that tires are 3.5%wt., while the other rubber goods are 1.6%wt. The dynamic development in the automotive industry increases the demand for new tires and, consequently, increases the number of waste tires. Estimated data showed that, each year, approximately 1000 million tires are not suitable for further use or retreading. Moreover, according to predictions, this value will increase by 20% by 2030 [5,6,7,8]. The current situation is a global environmental problem and a huge challenge for scientists and industry representatives.

At present, energy recovery is still the main method for resolving the issue of waste tires utilization. However, it should be pointed out that material recycling of waste tires is a more environmentally friendly option than their application as an alternative fuel in cement kilns or power plants [9].

During the recycling of waste tires, a common method is used in the form of shredding or grinding, which allows for the disintegration of waste tires into three fractions: steel, cord fibers, and ground tire rubber (GTR). The grinding of tires to a desired particle size distribution is usually performed at ambient temperature. GTR obtained by ambient grinding technology possesses a more developed surface as compared to grinding at cryogenic conditions, which positively affects further applications of GTR [10]. According to data that was presented by the European Tyre and Rubber Manufacturers Association (ETRMA) around 90% of waste tires in the European Union are recycled by this method [11].

Many research works confirmed that GTR can be successfully applied as a low-cost filler or modifier in polymers, bitumens, or concretes, and recent progress in this field was comprehensively described in review works [12,13,14,15,16]. However, usually increasing the content of GTR in a fresh matrix resulted in a deterioration of mechanical properties, which is related to the cross-linked structure of GTR and a rather poor compatibility with matrix. 

One promising method to improve matrix-GTR interactions is the devulcanization/reclaiming of GTR, which significantly improves processing (flowability) of waste rubber and improves the interfacial interactions on the phases boundary between GTR and matrix. 

Recent trends in this field research show that low-temperature devulcanization is gaining increasing attention [17,18,19]. The application of lower temperatures during processing allows for limiting the main chain scission and reducing the emission of volatile organic compounds that are characteristic for the reclaiming/devulcanization process. Furthermore, the main advantages of products based low-temperature devulcanization technologies are related to: (i) high quality (e.g., very good mechanical properties); (ii) less odors; and, (iii) reduced energy consumption.

On the other hand, some drawbacks are related to the processing of obtained products. This issue can be resolved by the application of plasticizers, such as bitumens [20] or petrochemical and renewable oils [21,22,23]. The application of thermoplastics is another possibility to enhance the processing of GTR and the performance of obtained materials [24,25]. 

Recently, Barbosa and Ambrósio [26] performed a thermo-mechanical devulcanization in the presence of 10%wt. of polypropylene or ethylene-vinyl acetate copolymer. The temperature that was used during extrusion was in the range of 210–270 °C. The authors indicated that a ethylene-vinyl acetate copolymer used during the reclaiming of natural rubber helps in the thermal stabilization of extrusion and it has a beneficial impact on the mechanical properties of the obtained product. 

Nunes et al. [27] investigated the effect of polypropylene or low-density polyethylene (in the range of: 15–25%wt.) on the thermo-mechanical devulcanization of GTR. The process was performed in a co-rotating twin-screw extruder at a temperature range of 220–270 °C. The results showed that polypropylene is more effective than low-density polyethylene during the devulcanization process and it promotes the formulation of reclaimed GTR with higher sol fraction.

Wang et al. [28] studied the impact of two compatibilizers: polyolefin elastomer (Engage™ 8180) and trans-polyoctenamer rubber (Vestenamer^®^ 8012) on the properties of recycled polyethylene/ground tire rubber (rPE/GTR) blends (with GTR content in the range of: 0–90%wt.). The samples were prepared in a co-rotating twin-screw extruder with a barrel temperature of between 150–180 °C. The results showed that, for rPE/GTR blend in ratio of 10/90% wt. with 9 phr of compatibilizer, tensile strength is ~2.1 MPa and elongation at break ~70% in the case of Vestenamer^®^ 8012, while, for Engage™ 8180, these parameters were ~0.9 MPa and ~72.5% (values estimated from graphs). 

Although, as presented above, thermoplastics were used to support the devulcanization process of GTR, according to the best of our knowledge there are no published data regarding their application in low-temperature devulcanization, which allows for better control of the process by the selective scission of cross-linking bonds. Moreover, the are no data about the impact of used thermoplastics on the possible recycling of GTR modified with a relatively small amount of thermoplastics (up to 10%wt.), while it seems that, in such systems, reclaimed rubber can be formed into the desired shape without the use of a curing system. 

In this paper, we propose a new method of GTR treatment by low-temperature extrusion in presence of two grades of ethylene-vinyl acetate copolymers. The modification process was performed in a co-rotating twin-screw extruder. The obtained products were compared with GTR that was modified with trans-polyoctenamer (tradename: Vestenamer^®^)—a commercial additive dedicated for waste rubber recycling. The processing and performance properties of obtained products have been investigated by the measurement of energy consumption, temperature of reclaimed GTR immediately after extrusion, melt flow index, Mooney viscosity, static mechanical properties, swelling behavior, and volatile organic compounds emission. Moreover, for the first time, the possibility of multiple processing of modified reclaimed GTR was also studied.

## 2. Experimental

### 2.1. Materials

In order to produce studied materials, the following components were used:

- Ground tire rubber (GTR)—obtained from passenger and truck tires, with particle sizes up to 0.4 mm, was received from Grupa Recykl S.A. (Śrem, Poland). The basic components of GTR are: natural rubber (NR), styrene-butadiene rubber (SBR), butadiene rubber (BR), additives (curing system, activators, plasticizers, etc.), carbon black, silica, and ash. Thermogravimetric analysis of GTR was presented in the work [29].
- Vestenamer^®^8012—semi-crystalline thermoplastic elastomer known as trans-polyoctenamer rubber (TOR) imported from the Evonik company (Essen, Germany). This additive acts as a plasticizer during rubber processing or reclaiming. TOR also reacts in the vulcanization process due to the presence of unsaturated bonds in the structure. It is characterized by a high compatibility with other materials.
- Sipchem EVA 2518—ethylene-vinyl acetate copolymer containing 18.2% vinyl acetate, manufactured by Sipchem (Khobar, Kingdom of Saudi Arabia).
- Escorene Ultra EVA FL00218—ethylene-vinyl acetate copolymer containing 18.0% vinyl acetate, obtained from Exxon Mobil Chemical (Machelen, Belgium).

Table 1 summarizes the basic properties of used modifiers provided by the manufacturers.

### 2.2. Sample Preparation

#### 2.2.1. Reclaiming of GTR Modified by Thermoplastics

Reclaimed GTR modified by thermoplastics was prepared using a co-rotating twin-screw extruder model EHP 2x20 Sline from Zamak Mercator (Skawina, Poland) with a L/d ratio of 40. The screw’s diameter was 20 mm and their rotational speed was equal to 250 rpm. The extruder was equipped with eleven heating zones. The temperatures in individual heating zones (from hopper to extrusion die) on the barrel of the extruder were: 40/40/60/60/60/60/60/60/60/60/60 °C. Both components, GTR and 10 phr of thermoplastics (TOR-Vestenamer^®^8012, EVA1—Sipchem EVA 2518 or EVA2 - Escorene Ultra EVA FL00218), were directly introduced into the hopper with a constant feeding rate (2.5 kg/h) that was provided by a gravimetric feeding system from Hydrapress Sp. z o.o. (Białe Błota, Poland). 

**Figure 1:**
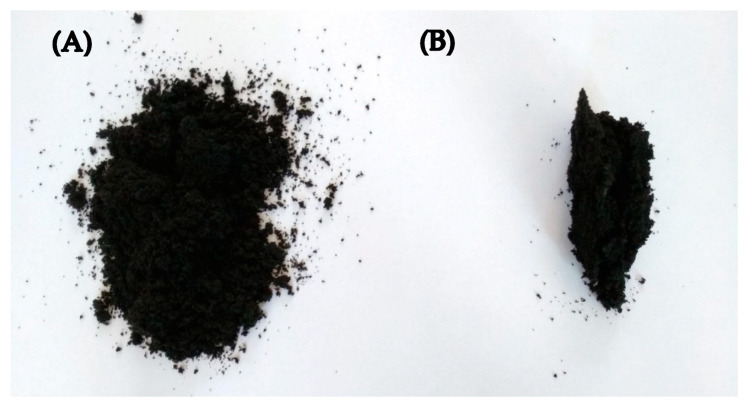
The appearance of products prepared by low-temperature extrusion: (**A**) ground tire rubber (GTR) and (**B**) GTR in presence of thermoplastics.

The extrusion resulted in partial reclaiming of GTR in the presence of thermoplastics, as presented in Figure 1. The appearance of modified reclaimed GTR was similar, regardless of the thermoplastic type. 

#### 2.2.2. Formulation of Modified Reclaimed GTR by Compression Molding

Modified reclaimed GTR was directly formulated into sheets by compression molding for 5 min. under a pressure of 4.9 MPa. In order to better understand the effect of molding temperature on the performance of obtained materials, modified reclaimed GTR samples were compression molded at three different temperatures: 140, 160, and 180 °C. To clarify, Table 2 summarizes the samples formulation conditions and coding. The samples were coded as GTR+X-Y, where X is used thermoplastics (TOR, EVA1, or EVA2), while Y is compression molding temperature (140, 160, or 180 °C).

### 2.3. Measurements

Energy consumption during the extrusion of GTR was evaluated by two methods. The first method is based on direct measurement of energy consumption by electricity meter (include all items in the extruder, but the main energy consumption is related to barrel heaters and drive motor). The second method determines specific mechanical energy (SME, in kWh/kg—based on energy consumption of drive motor), which was calculated while using Equation (1):

(1) $$\mathrm{SME}=\frac{N}{Q}$$

where: N is the consumption of drive motor power (kW) and Q is a throughput (kg/h).

Temperature distribution in GTR that was treated during extrusion was measured using an infrared thermal imaging camera model Testo 872 (Testo SE & Co. KGaA, Lenzkirch, Germany). Table 3 presents the specification during measurements performed by Testo 872.

The melt flow index of samples was investigated using the Zwick mFlow plastometer (ZwickRoell Group, Ulm, Germany), according to ISO 1133 at 210 °C, with a load of 10 kg.

Mooney viscosity of modified GTR was investigated while using MV 2000 viscometer (Hudson, OH, USA) according to ISO 289 at 125 °C. During measurements, a large rotor was used. The preheating time was 1 min and the measurement time was 4 min. At least three tests were performed for each sample. 

The tensile strength and elongation at break of the obtained modified reclaimed rubbers were tested at room temperature according to ISO 37. Tensile tests were carried out on the universal Zwick/Roell Z020 testing machine (ZwickRoell Group, Ulm, Germany) at a constant speed of 500 mm/min. Extensometers were used to exactly measure relative elongation at break of the samples. At least five measurements were performed for each sample. 

Shore hardness type A was measured with a Zwick 3130 durometer (ZwickRoell Group, Ulm, Germany) in accordance with the standard ISO 7619-1. The reported results are the means of ten measurements per sample. 

The density of the samples was measured by the Archimedes method in accordance with ISO 2781 while using an analytical balance model AS 110.R2 from Radwag (Radom, Poland). The test was carried out at room temperature and it consisted of weighing the material in air and then in a methanol medium. The density value quoted in the paper is the average of at least three measurements per sample.

The determination of the swelling degree of the modified GTR and its cross-link density was carried out on the basis of the equilibrium swelling method. Small pieces of samples weighing approximately equal to 0.2 g were immersed in toluene at room temperature for 72 h. The swelling degree was calculated according to Equation (2):

(2) $$Q=\frac{m_{t}-m_{0}}{m_{0}}\times 100\%$$

where: Q—swelling degree (%); m_t_—a mass of the sample swollen after time (g); m_0_—an initial mass of the sample (g).

The sol fraction was determined on the basis of the mass difference of the initial sample and the dried sample after extraction according to Equation (3). The remaining part is a gel fraction (4):

(3) $$F_{\mathrm{sol}}=\frac{m_{0}-m_{k}}{m_{0}}\times 100\%$$

(4) $$F_{\mathrm{gel}}=100\%-F_{\mathrm{sol}}$$

where: F_sol_—the content of sol fraction (%); F_gel_—the content of gel fraction (%); m_0_—an initial mass of the sample (g), and m_k_—a mass of the dried sample after extraction (g).

Cross-link density was determined according to the Flory–Rehner Equation (5) [30]:

(5) $$v_{e}=\frac{-\left[\ln\left(1-V_{r}\right)+V_{r}+{\chi V}_{r}^{2}\right]}{\left[V_{1}\left(V_{r}^{\frac{1}{3}}-\frac{V_{r}}{2}\right)\right]}$$

where: ν_e_—cross-link density (mol/cm^3^); V_r_—gel volume in the swollen sample (cm^3^); V_1_—solvent molar volume (toluene = 106.2 cm^3^/mol), χ—polymer-solvent interaction parameter (in the calculations, it was assumed to be 0.391).

The Flory–Rehner equation is correct for non-filled compounds. The presence of rubber waste in the examined materials causes them to have a high content of carbon black. Therefore the Kraus correction should be included in order to calculate the actual cross-link density. However, in the present study, this correction was omitted, assuming that the filler content in all of the tested samples is the same, which allows for comparison of the obtained values with each other, as suggested by Barbosa and Ambrósio [26]. 

Total volatile organic compounds (TVOCs) parameter and chemical structure of volatile organic compounds emitted from the modified GTR were determined using microscale stationary emission chamber—Markes Micro-Chamber/Thermal Extractor™—μ-CTE™ 250 (Markes International Ltd., Llantrisant, UK) in which 2.5–3.0 g samples were conditioned for 20 min. at 40 °C. Volatile organic compounds emitted from the modified GTR were collected using stainless steel tubes filled with Tanax TA adsorption bed ((Markes International Ltd., Llantrisant, UK) under the influence of a carrier gas (nitrogen) at a rate of 25 mL/min. Subsequently, the adsorbed analytes were released through a two-stage process of thermal desorption (Markes Series 2 Thermal Desorption Systems; UNITY/TD100, Llantrisant, UK). Subsequently, quantitative and qualitative analysis of analytes that were released from the modified GTR were performed on: GC-FID system (Agilent 7820A GC, Agilent Technologies, Inc., Santa Clara, CA, USA) and GC-MS system (GC Agilent Technologies 6890 and 5873 Network Mass Selective Detector; Agilent Technologies, Inc., Agilent, Santa Clara, CA, USA). More detailed information regarding used equipment and methodology are presented in works [31,32,33].

## 3. Results and Discussion

### 3.1. Temperature and Energy Consumption Measurements

**Figure 2:**
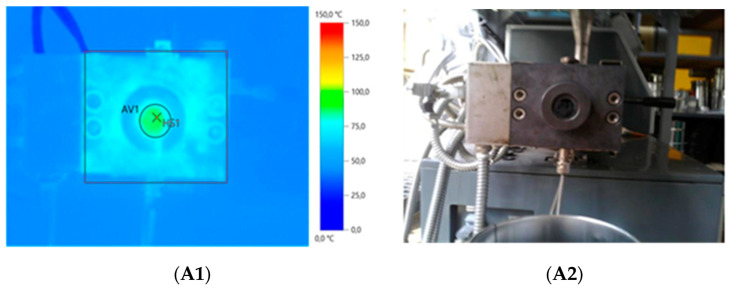

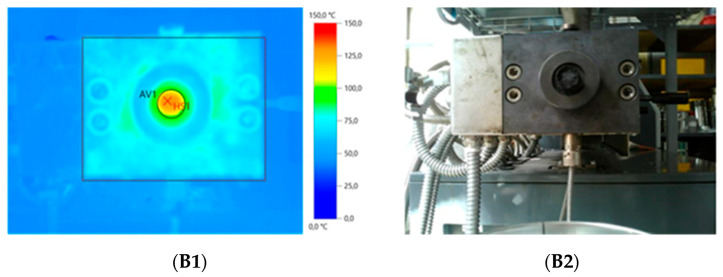
IR camera images of: GTR (**A1**,**A2**) and GTR+EVA2 (**B1**,**B2**).

It was observed that relatively small amounts of thermoplastics (10 phr) during low temperature devulcanization allow for the preparation of modified GTR in form of solid profiles (see Figure 1). Thermoplastics act like a binder that connects GTR particles, which strongly affects the temperature of the material after extrusion. Figure 2 presents the temperature distribution of GTR on the die (Figure 2(A1,A2)) and GTR+EVA2 (Figure 2(B1,B2)) measured by the thermograph IR camera immediately after extrusion.

The results showed that, for pure GTR, the maximal temperature (T_Maximal_) after extrusion was 69.0 °C, while the average temperature (T_Average_) was 63.0 °C. The addition of thermoplastics resulted in a significant increase in the average and maximal temperature of reclaimed GTR. Depending on the thermoplastics used, T_Average_ was 107.4, 110.4, and 102.9, while T_maximal_ was 136.1, 126.9, and 120.3 for GTR+TOR, GTR+EVA1, and GTR+EVA2, respectively.

The evaluation of energy consumption during processing provides useful information about process efficiency and determine the possibility of its application at an industrial scale. A common practice is to measure specific mechanical energy (SME), related to power necessary for driver motor. During presented research, this method was extended by the application of an electricity meter installed in the extruder. In this solution, energy consumption is determined for all of the items in the extruder, but most power is related to the driver motor and barrel heaters. Table 4 shows the results of temperature measurements and energy consumption. 

Diaz et al. [34] performed thermo-mechanical reclaiming of ethylene-propylene-diene rubber in a special high shear mixer (HSM) designed as a rotor-stator system that was equipped with a cooling system dedicated independently to the rotor and stator to control rubber self-heating phenomenon. In conclusion, the authors indicated that specific mechanical energy can be used as an indicator of rubber reclaiming efficiency. It was observed that the energy needed for the waste rubber treatment decreased with a lower particle size of waste rubber. This trend is also visible in the presented work. The application of thermoplastics resulted in the formulation of a solid profile which significantly increases SME (0.423–0.489 kWh/kg) when comparing to GTR (0.142 kWh/kg) in form of powder with the developed surface, as presented in Table 4. More interesting is the tendency observed for total energy consumption (measured for all items in the extruder, but the main source of power consumption are motor driver and heaters) and percentage content of SME in total energy. The results showed that the application of a relatively small amount of thermoplastics (10 phr) in modified GTR resulted in 24.7–35.0% increase of total energy consumption when compared to GTR (an increase from 0.420 kWh/kg to 0.524–0.567 kWh/kg—depending on used thermoplastic). It was noticed that the GTR percentage content of SME in total energy was 33.9%, while the addition of thermoplastics resulted in an increase of this parameter to values in the range: 80.7–86.3%. This is related to the self-heating phenomenon of GTR with thermoplastics during extrusion, which is caused by increased friction due to the presence of a thermoplastic modifier. It significantly reduces the energy consumption of barrel heaters.

### 3.2. MFI and Mooney Viscosity Measurements

The MFI of used thermoplastics at 190 °C and 2.16 kg was 13.8 g/10 min. for TOR; 2.5 g/10 min. for EVA1 and 1.7 g/10 min. for EVA2, as presented in Table 1. For modified GTR, the MFI test conditions at 210 °C with load 10 kg (preliminary studies were performed for temperature in the range: 190–210 °C, load: 2.16–10 kg) were used in order to investigate processing properties. In studied conditions, prepared samples have not shown adequate flowability and, therefore, the MFI parameter could not be determined. Samples of modified GTR showed rubber-like behavior with limited flow. During measurement, the ground particles of modified GTR (particle dimension suitable to apply in a plastometer barrel) have merged due to elevated temperature and pressure. This confirms that the pressure is too low to enable the modified GTR to pass through a standard capillary die with a diameter of 2.095 mm. Garcia et al. [35] measured the melt-viscosity of reclaimed rubber using a high-pressure capillary rheometer with a capillary die diameter of 1mm and L/D ratio of 20. The measurement temperature was 190 °C and the shear rate was in the range of: 300–15000 s^−1^. The authors pointed out that the possibility to determine the viscosity of studied samples was strongly correlated with the devulcanization degree of the sample. The results showed that this method cannot be applied for reclaimed GTR characterized by 83.5% of gel fraction content (the viscosity was measured for samples with gel fraction content lower than 73.0%). The products that are characterized in this research are characterized by a higher value of this parameter (see Table 5).

Taking into account the above-mentioned examples, it seems that the only way to test the reclaimed rubber with a capillary rheometer or plastometer is to change the standard dimensions of the die, which should be considered during future studies in this field.

During studies, the Mooney viscosity of modified GTR was also investigated and the results are summarized in Table 4. Regardless of the type of used thermoplastics, it was found that the application of thermoplastics allows for the determination of viscosity of modified reclaimed GTR by Mooney viscometer, while measurements for unmodified GTR were not possible. Mooney viscosity —ML(1+4) 125 °C for studied samples were: 124.6 MU for GTR+TOR; 150.6 MU for GTR+EVA1; and, 142.3 MU for GTR+EVA2. The lowest value was determined for the GTR+TOR sample, which is related to the highest MFI value of this thermoplastic modifier (see Table 1). For EVA copolymers, the viscosity of reclaimed GTR modified by EVA1 was slightly higher when compared to reclaimed GTR with EVA2. In this case, there is no simple correlation between MFI of EVA copolymers and Mooney viscosity of reclaimed GTR modified by EVA. This indicates that slight differences between sample GTR+EVA1 and GTR+EVA2 can be related to the complex composition of GTR and its intermolecular interactions with EVA copolymer, which also affect the temperature of material after extrusion (see Table 4).

### 3.3. Swelling Behavior

**Figure 3:**
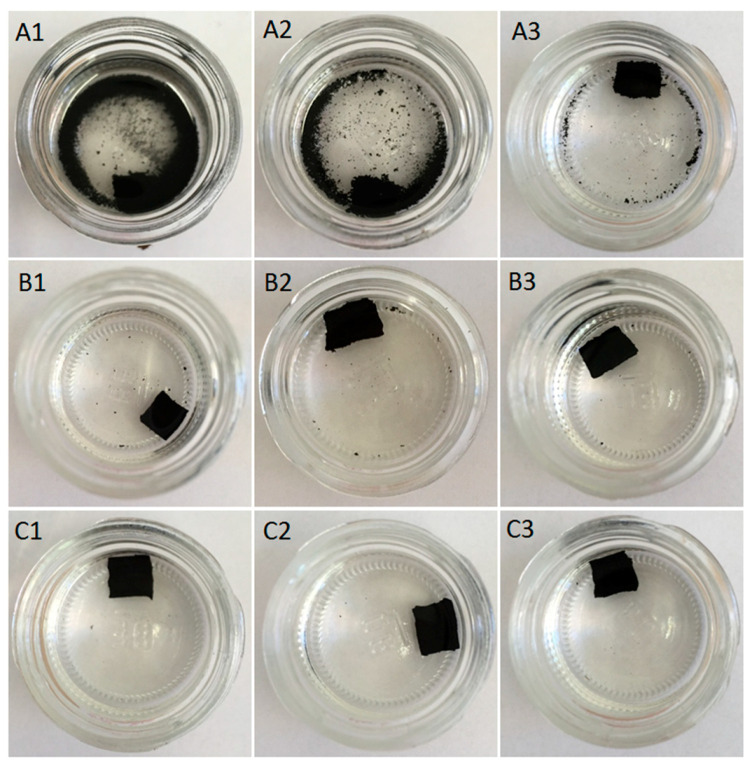
Samples after a few minutes of extraction in toluene: (**A1**) GTR+TOR-140 °C; (**A2**) GTR+TOR-160 °C; (**A3**) GTR+TOR-180 °C; (**B1**) GTR+EVA1-140 °C; (**B2**) GTR+EVA1-160 °C; (**B3**) GTR+EVA1-180 °C; (**C1**) GTR+EVA2-140 °C, (**C2**) GTR+EVA2-160 °C, and (**C3**) GTR+EVA2-180 °C.

**Figure 4:**
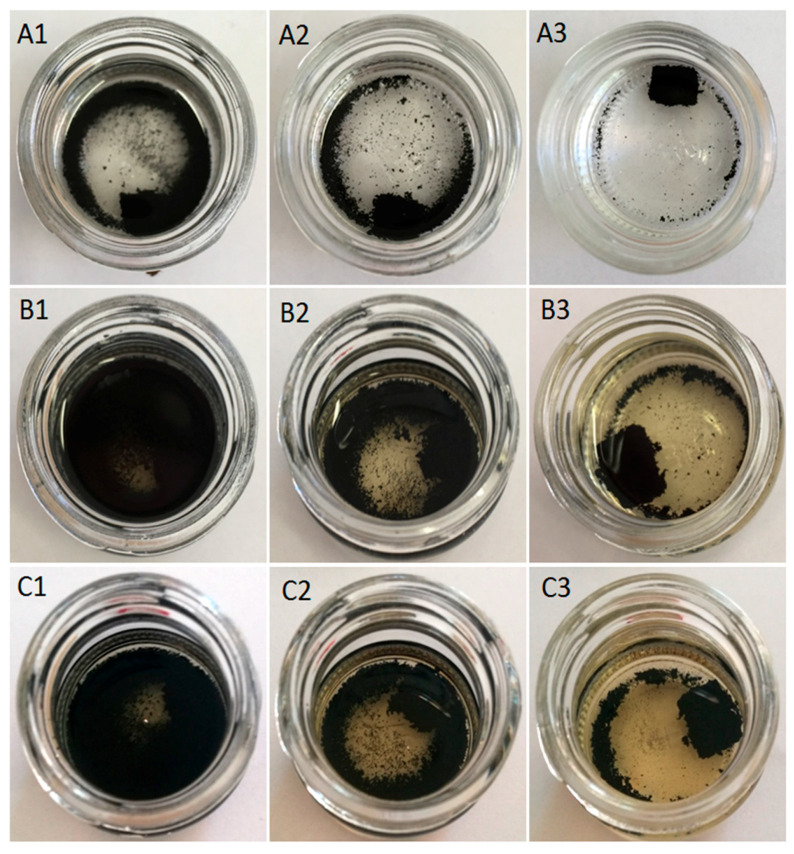
GTR modified with trans-polyoctenamer rubber (TOR); the letters indicate the extraction time in toluene: (**A**) few minutes; (**B**) 24 h; (**C**) 72 h; the numbers indicate the temperature at which the samples were compression molded: (**1**) 140 °C; (**2**) 160 °C; (**3**) 180 °C.

**Figure 5:**
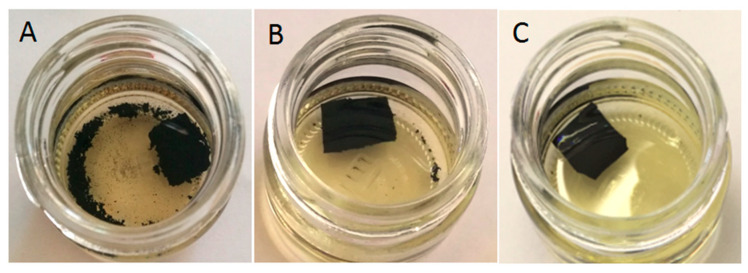
Samples after 72 h extraction in toluene: (**A**) GTR+TOR-180 °C; (**B**) GTR+EVA1-180 °C; (**C**) GTR+EVA2-180 °C.

The swelling behavior of GTR that was modified by thermoplastics was investigated in toluene at room temperature for 72 h. The appearance of studied samples as a function of time was presented in Figure 3, Figure 4 and Figure 5. As can be observed in Figure 3(A1–A3), the sample coded as GTR+TOR showed very low toluene resistance. GTR+TOR samples start to disintegrate after a few minutes of solvent extraction. The resistance for toluene increased with higher temperature of sample compression, in the order: GTR+TOR-180 °C > GTR+TOR-160 °C > GTR+TOR-140 °C, as presented in Figure 4.

As presented in Figure 5, samples GTR+EVA1 and GTR+EVA2 were stable after 72 h extraction, which allowed for the determination of their swelling degree, cross-link density, sol, and gel fraction presented in Table 5. For comparison, the results for GTR compressed in the same temperatures are also presented. It was found that for GTR modified with thermoplastics swelling degree is in the range of 172–184%, cross-link density: 1.29–1.49 mol/cm^3^ · 10^−4^; sol fraction: 9.4–11.0% (gel fraction: 89.0–90.6%). The addition of EVA copolymers resulted in the decrease of cross-link density and increase of swelling degree as compared to unmodified GTR, while the effect of this modifier on sol and gel fraction was negligible. Moreover, the presented results indicate that the cross-link density of the material is slightly higher for GTR+EVA2 when comparing to GTR+EVA1. This confirms that the lower temperature of the material after extrusion (see Table 4) resulted in a lower devulcanization degree of GTR during processing, which affects the matrix-filler interactions [36,37,38]. 

The analysis of swelling behavior in relation to changes in the sintering temperature shows that, as the temperature of this process increases, the degree of swelling increases and the cross-linking density and the sol fraction decrease. This is due to the fact that, with the increase in temperature, a partial reclaiming/degradation of the GTR takes place during the preparation of revulcanizates. 

### 3.4. Physico-Mechanical Properties

Table 6 summarizes the physico-mechanical properties of GTR modified by thermoplastics. For comparison, the results for GTR formulated in the same conditions (pressure, temperature) as GTR modified by thermoplastics are also presented in Table 7. It was found that a relatively small amount of EVA copolymers (regardless of EVA copolymer grade) increased tensile strength by 28%, elongation at break by 98%, and hardness by 12% as compared to values determined for unmodified GTR. Moreover, a slight decrease of density of samples GTR+EVA1 and GTR+EVA2 was observed in comparison to GTR, which is obviously related to the lower density of the thermoplastics when compared to GTR. 

The results showed that temperature had a significant impact on the values of studied physico-mechanical properties. Usually, the tensile properties increased with higher compression temperature. However, there is no simple correlation between all studied samples. For example, surprisingly, sample GTR+TOR compressed at 140 °C showed the lowest values of tensile strength and elongation at break among studies samples. This indicates that, for this system, 140 °C was too low to allow efficient encapsulation of GTR by TOR. Furthermore, GTR devulcanization during compression was rather limited in this temperature, which also affects the final performance properties of the obtained materials.

In Table 7, the final properties of modified GTR were compared with other EVA/GTR systems characterized by other research groups. The literature data showed that EVA/GTR systems prepared via melt-blending in ratio 90/10%wt. and 60/40%wt. are characterized by tensile strength in the range ~6–9 MPa, elongation at break ~310–550%, and hardness ~34–81 (Sh A) [39,40,41], while, for LDPE/GTR/EVA with 40–45%wt. content of GTR [42,43] tensile strength and elongation at break values are ~5–7.8 MPa and ~180–185%, respectively. The presented parameters are higher than those determined for studied materials. However, it should be pointed out that the obtained materials only contain 10 phr (9%wt.) of fresh EVA, while the systems described in the literature are usually modified with 60–90%wt. of thermoplastics. Moreover, analysis of literature data showed that the tensile strength of obtained materials is significantly higher than the 2.0 MPa determined for reclaimed GTR/EVA system in the ratio of 70/30%wt. [44]. Finally, GTR/rPE/TOR 90/10/9 proposed by a different research group [28] shows tensile strength at 2.1 MPa, elongation at break at approx. 70%, and hardness at approx. 78 Sh A, giving lower values than those that are presented in our study. 

**Table 7:** Tensile properties of EVA/GTR systems described in literature.

Sample Composition	Sample Preparation	Tensile Strength (MPa)	Elongation at Break (%)	Hardness (Sh A)	References
GTR/EVA 100/10	extrusion at 60 °C, compression molding at 140–180 °C	2.7–3.4	125–164	63–65	This study
GTR/recycled PE/TOR 90/10/9	extrusion at 150–180 °C injection molding at 180–190 °C	~2.1	~70	~78	[28]
EVA/GTR 90/10	batch mixer at 140 °C, compression molding at 123 °C	~9*	~550 *	~34 *	[39,40]
EVA/GTR 60/40	batch mixer at 140 °C, compression molding at 123 °C	~6*	~310 *	~81 *	[41]
LDPE/GTR/EVA 35/45/20	extrusion at 165–175 °C, injection molding at 165–190 °C	~5^*^	~185 *	-	[42]
recycled LDPE/GTR/EVA 30/40/30	extrusion at 165–175 °C, injection molding at 165–190 °C	~7.8*	~180 *	-	[43]
Reclaimed GTR/EVA 70/30 and 50/50	batch mixer at 120 °C, compression molding at 130 °C	2.0–5.4	300–999	72–80	[44]

* The value estimated from graphs.

### 3.5. Evaluation of Modified Reclaimed GTR Recycling Possibility 

**Figure 6:**
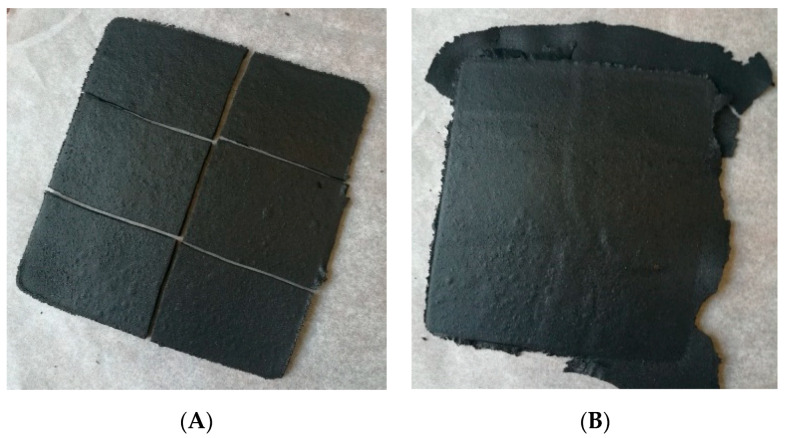
Appearance of GTR+EVA2 sample: (**A**) reference sample; (**B**) sample after material recycling (sample compressed in the same conditions as the reference sample).

During this study, for the first time, the possibility of modified reclaimed GTR recycling was investigated. The recycling of the GTR+EVA2 sample was simulated by multiple processing (three rounds) in the same conditions (temperature, pressure, and time of compression molding) as a reference sample. The appearance of the sample before and after material recycling is presented in Figure 6. The changes in tensile strength, elongation at break, and hardness as a function of the recycling cycle were studied and the results are summarized in Table 8. The results showed that reclaimed GTR that is modified by EVA copolymers can be successfully recycled without significant deterioration of tensile properties after three cycles of material recycling.

### 3.6. Determination of Volatile Organic Compounds 

Two types of analyses were performed in order to determine the emission of volatile organic compounds emitted from modified GTR: gas chromatography with a flame ionization detector (GC-FID) and gas chromatography with mass spectrometry (GC-MS). GC-FID study allows for the investigation of the total volatile organic compounds (TVOCs) emitted from the modified reclaimed rubbers, while the GC-MS analysis provides information about the chemical structure of volatile organic compounds released from prepared materials. Table 9 presents the results of TVOCs analysis for studied samples. It was observed that a relatively small amount of thermoplastics in GTR limits the emission of volatile organic compounds. TVOCs parameter for GTR modified by thermoplastics decreased by 81–105% when comparing to GTR (decrease from 1.56 μg/g to 0.76–0.86 μg/g). This phenomenon is related to the higher thermal stability of thermoplastics when comparing to GTR, which is very important during high-temperature processing via extrusion, injection, or compression molding. For example, Ramarad et al. [44] studied the thermal stability of reclaimed GTR/EVA blends by thermogravimetric analysis. The results indicated that volatile compounds content in reclaimed GTR is 8.25%wt., while for reclaimed GTR modified by 30%wt. of EVA volatile compounds content decrease to 5.40%wt.

Table 10 summarizes chemical structures of volatile organic compounds emitted from GTR+EVA2, as determined by GC-MS analysis. The presented results showed that the investigated volatile organic compounds are mostly oxidative degradation products of GTR and EVA. For GTR, determined VOCs can be divided into three groups: (i) residual vulcanization accelerators (benzothiazole, aniline); (ii) natural rubber degradation products (1-butanol; methyl isobutyl ketone; cyclohexanone; cyclooctane); and, (iii) styrene-butadiene rubber degradation products (toluene; xylene; styrene; cyclohexanone; cyclooctane; benzaldehyde; acetophenone). Low molecular compounds that are related to the degradation of EVA are: butanoic acid, ethyl ester; 5-methyl-2-hexanone and 1-butanol, 3-methyl-, acetate. 

The highest intensity of peak area was noticed for limonene (13.25%) and benzothiazole (9.87%), which indicates that these two volatile organic compounds should be considered as markers for GTR reclaiming progress. Limonene concentration provides information regarding natural rubber oxidative degradation level. Due to fact that natural rubber is more prone to thermal degradation than synthetic rubbers, limonene concentration is correlated to the level of main chain scission in GTR. On the other hand, the benzothiazole level gives information about vulcanization accelerators present in GTR. Therefore, benzothiazole concentration can be a useful indicator of the disintegration of a three-dimensional network that is formed during sulfur vulcanization (degree of devulcanization). 

## 4. Conclusions

Regardless of the proposed methodology, GTR recycling must be an economically viable and environmentally acceptable process. This means that it must be possible to use the waste material without the need for a selective collection of post-consumer tires, the treatment and modification must be cost-effective, and the products cannot be characterized as hazardous. The recycling and modification methods proposed in the study, as well as the characteristics of the finished products, are part of a pro-ecological approach to the subject. In the paper, GTR was modified by low-temperature extrusion in the presence of two grades of ethylene-vinyl acetate copolymers and trans-polyoctenamer—commercially available additive dedicated for waste rubber recycling. The reclaiming process was analyzed in terms of energy consumption, the temperature generated during self-heating of rubber, melt flow index, and Mooney viscosity. The modified reclaimed GTR samples were reactively sintered under three different temperatures (140, 160, and 180 °C). Subsequently, the samples were analyzed for static mechanical properties, swelling behavior, and VOC emissions. In addition, the possibility of recycling already received revulcanizates was investigated, which is of key importance in terms of the “green” approach. The results showed that the application of 10 phr of thermoplastic facilitates the self-heating phenomenon of GTR, reducing the energy consumption related to heating of barrels (SME of pure GTR—33.9%, SME of modified GTR—80.7–86.3%), while the overall energy consumption was higher (0.420 kWh/kg for GTR and 0.567, 0.545, and 0.524 kWh/kg for GTR+TOR, GTR+EVA1, and GTR+EVA2, respectively). The addition of thermoplastics reduced the amount of VOCs emitted to the atmosphere by 81–105%, which will significantly increase the possibilities for practical use of GTR in industrial applications. Moreover, obtained samples (GTR+EVA1 and GTR+EVA2) were characterized with satisfactory mechanical properties (tensile strength—2.7–3.4 MPa, elongation at break—125–164%, and hardness—63–65 Sh A) and recyclability without significant mechanical property loss per each stage. 

Future research in this field should focus on: (i) use of cross-linking systems, plasticizers, or other thermoplastic modifiers to improve processing and tensile parameters, (ii) analysis of in-process energy consumption and evaluation of its reduction possibilities, and (iii) analysis of VOCs generated during processing and assessment of their impact on the environment and human health.

## Figures and Tables

**Table 1:** Properties of the used thermoplastic modifiers.

Properties	Additive		
	Vestenamer^®^8012 *	Sipchem EVA 2518 *	Escorene Ultra EVA FL00218 *
Abbreviation	TOR	EVA1	EVA2
Density at 25 °C, g/cm^3^	0.910	0.935	0.940
MFI_190 °C, 2.16 kg_, g/10 min	13.8	2.5	1.7
Vicat softening temperature, °C	-	64	62
Melting temperature, °C	54	87	87

* Information from technical data sheets provided by producers.

**Table 2:** Sample formulation and coding.

Sample Composition *	Compression Molding Temperature (°C)	Sample Coding
GTR + 10 phr Vestenamer^®^8012	140	GTR+TOR-140 °C
	160	GTR+TOR-160 °C
	180	GTR+TOR-180 °C
GTR + 10 phr Sipchem EVA 2518	140	GTR+EVA1-140 °C
	160	GTR+EVA1-160 °C
	180	GTR+EVA1-180 °C
GTR + 10 phr Escorene Ultra EVA FL00218	140	GTR+EVA2-140 °C
	160	GTR+EVA2-160 °C
	180	GTR+EVA2-180 °C

* All samples were prepared in the same conditions.

**Table 3:** Specification and measurement parameters of Testo 872.

Item	Specification *
Measuring range	−30 to 650 °C
Accuracy	± 2 °C/±2%
Infrared resolution	320 × 240
Thermal sensitivity	60 mK
Geometric resolution	2.3 mrad
SuperResolution	640 × 480 pixels/1.3 mrad
IR image refresh rate	9 Hz
Spectral range	7.5–14 μm

* According to producer data.

**Table 4:** Temperature and energy consumption measurements as function of sample composition.

Sample Composition	Physical form of Extruded Material	IR Camera Record		Energy Consumption (kWh/kg)			Mooney Viscosity ML(1+4) 125 °C (MU)
		T_Average_ (°C)	T_Maximal_ (°C)	SME	Total *	SME in Total Energy (%)	
**GTR**	**Powder with developed surface**	63.0	69.0	0.142	0.420	33.9	- **
GTR+TOR	Solid profile	107.4	136.1	0.489	0.567	86.3	124.6 ± 0.6
GTR+EVA1	Solid profile	110.4	126.9	0.457	0.545	83.8	150.6 ± 1.7
GTR+EVA2	Solid profile	102.9	120.3	0.423	0.524	80.7	142.3 ± 0.3

* Measured directly by electricity meter installed in extrusion line. ** The viscosity of sample cannot be determined in studied conditions.

**Table 5:** Swelling degree, cross-link density, sol, and gel fraction of GTR modified by ethylene vinyl acetate (EVA).

Sample Coding	Swelling Degree (%)	Cross-Link Density (mol/cm^3^10^−4^)	Sol Fraction (%)	Gel Fraction (%)
GTR-140 °C	166 ± 2	1.64 ± 0.06	9.6 ± 0.1	90.4
GTR-160 °C	163 ± 1	1.65 ± 0.01	9.8 ± 0.1	90.2
GTR-180 °C	169 ± 4	1.55 ± 0.07	10.5 ± 0.3	89.5
GTR+EVA1-140 °C	178 ± 3	1.36 ± 0.02	11.0 ± 0.8	89.0
GTR+EVA1-160 °C	180 ± 1	1.34 ± 0.01	10.9 ± 0.2	89.1
GTR+EVA1-180 °C	184 ± 1	1.29 ± 0.04	10.6 ± 0.3	89.4
GTR+EVA2-140 °C	172 ± 3	1.53 ± 0.06	9.4 ± 0.2	90.6
GTR+EVA2-160 °C	174 ± 1	1.49 ± 0.02	9.6 ± 0.2	90.4
GTR+EVA2-180 °C	178 ± 1	1.40 ± 0.01	10.1 ± 0.1	89.9

**Table 6:** Physico-mechanical properties of GTR and GTR modified by thermoplastics.

Sample Coding	Tensile Strength (MPa)	Elongation at Break (%)	Hardness (Sh A)	Density (g/cm^3^)
GTR-140 °C	2.4 ± 0.2	77 ± 7	59 ± 1	1.170 ± 0.002
GTR-160 °C	2.4 ± 0.2	71 ± 6	55 ± 1	1.163 ± 0.006
GTR-180 °C	2.6 ± 0.1	79 ± 4	57 ± 1	1.149 ± 0.007
GTR+TOR-140 °C	1.7 ± 0.2	49 ± 11	63 ± 1	1.090 ± 0.007
GTR+TOR-160 °C	2.1 ± 0.2	73 ± 11	64 ± 1	1.121 ± 0.001
GTR+TOR-180 °C	2.4 ± 0.2	92 ± 14	65 ± 1	1.125 ± 0.003
GTR+EVA1-140 °C	2.7 ± 0.4	125 ± 13	64 ± 1	1.130 ± 0.003
GTR+EVA1-160 °C	3.1 ± 0.1	138 ± 10	64 ± 1	1.123 ± 0.003
GTR+EVA1-180 °C	3.4 ± 0.4	164 ± 14	64 ± 1	1.127 ± 0.003
GTR+EVA2-140 °C	3.4 ± 0.5	147 ± 16	65 ± 1	1.140 ± 0.004
GTR+EVA2-160 °C	3.2 ± 0.5	146 ± 11	63 ± 1	1.132 ± 0.002
GTR+EVA2-180 °C	3.2 ± 0.5	151 ± 21	63 ± 1	1.134 ± 0.003

**Table 8:** Tensile properties of GTR+EVA2 sample as function of recycling cycles.

Sample Coding	Tensile Strength (MPa)	Elongation at Break (%)	Hardness (Sh A)
Reference	3.2 ± 0.5	146 ± 11	63 ± 1
1st round of recycling	2.9 ± 0.4	136 ± 17	63 ± 1
2nd round of recycling	3.1 ± 0.4	143 ± 17	64 ± 1
3rd round of recycling	2.7 ± 0.5	123 ± 25	63 ± 1

**Table 9:** Total volatile organic compounds (TVOCs) parameter determined for GTR and GTR modified by thermoplastics.

Sample Coding	TVOCs (μg/g)
GTR-180 °C	1.56
GTR+TOR-180 °C	0.86
GTR+EVA1-180 °C	0.76
GTR+EVA2-180 °C	0.78

**Table 10:** Volatile organic compounds identified by gas chromatography with mass spectrometry (GC-MS) measurement for GTR modified with ethylene-vinyl copolymers.

Retention Time (min)	Identified Compound	Chemical Structure	Molecular Weight (g/mol)	Peak Area (%)	Match Quality (%)	Source	References
6.58	1-butanol	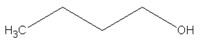	74.12	2.68	90	natural rubber present in GTR	[45]
7.92	methyl-isobutyl ketone	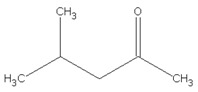	100.16	4.74	87	natural rubber and anti-aging agents present in GTR	[18,45,46]
8.64	toluene	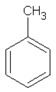	92.14	0.84	92	styrene-butadiene rubber present in GTR	[47,48]
8.94	butanoic acid, ethyl-ester	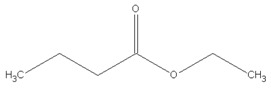	116.16	2.86	89	EVA	-
10.09	5-methyl-2-hexanone	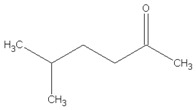	114.19	1.62	91	EVA	-
10.37	1-butanol, 3-methyl-, acetate	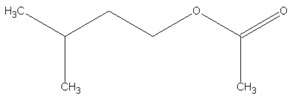	130.18	4.67	90	EVA	-
10.59	xylene	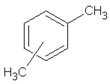	106.16	0.58	94	styrene-butadiene rubber present in GTR	[47,48]
11.06	styrene	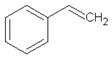	104.15	1.27	95	styrene-butadiene rubber present in GTR	[47,48]
11.19	cyclohexanone	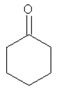	98.14	5.33	91	elastomers present in GTR	[48]
11.80	cyclooctane	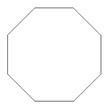	112.21	4.95	97	elastomers present in GTR	-
12.50	benzaldehyde	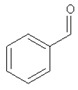	106.12	1.43	96	styrene-butadiene rubber present in GTR	[48]
12.70	aniline	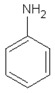	93.13	2.30	95	vulcanization accelerators present in GTR	[45]
13.58	limonene	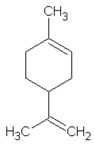	136.23	13.25	94	natural rubber present in GTR	[45,49]
14.36	acetophenone	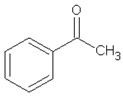	120.15	1.13	95	styrene-butadiene rubber present in GTR	-
17.37	benzothiazole	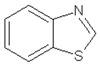	135.19	9.87	95	vulcanization accelerators present in GTR	[18,45,47,48]
